# APOE genetic variability in an Egyptian cohort of PD

**DOI:** 10.3389/fnins.2025.1579968

**Published:** 2025-05-23

**Authors:** Eman M. Khedr, Martina B. William, Aliaa A. Elhosseiny, Ali Shalash, Gharib Fawi, Mohamed H. Yousef, Shaimaa El-Jaafary, Hamin Lee, Alina Jama, Mohamed Koraym, Asmaa Helmy, Yara Salah, Peter George, Nourelhoda A. Haridy, Samir Nabhan, Agsha Atputhavadivel, Sara Elfarrash, Gaafar Ragab, Mohamed Tharwat Hegazy, Yasmin Elsaid, Asmaa S. Gabr, Nourhan Shebl, Lobna Aly, Nesreen Abdelwahhab, Tamer M. Belal, Nehal A. B. Elsayed, Mohamed El-Gamal, Shimaa Elgamal, Salma Ragab, Jaidaa Mekky, Henry Houlden, Mie Rizig, Mohamed Salama

**Affiliations:** ^**1**^Department of Neurology, Faculty of Medicine, Assiut University, Assiut, Egypt; ^2^Institute of Global Health and Human Ecology, The American University in Cairo, Cairo, Egypt; ^3^Department of Clinical Pharmacy, Faculty of Pharmacy, Assiut University, Assiut, Egypt; ^4^Department of Pharmacology and Toxicology, Faculty of Pharmacy, The British University in Egypt, Cairo, Egypt; ^5^Department of Neurology, Faculty of Medicine, Ain Shams University, Cairo, Egypt; ^6^Department of Neurology, Faculty of Medicine, Sohag University, Sohag, Egypt; ^7^Department of Neurology, Faculty of Medicine, Cairo University, Cairo, Egypt; ^8^Department of Neuromuscular Diseases, UCL Queen Square Institute of Neurology, UCL, London, United Kingdom; ^9^Department of Medical Physiology, Faculty of Medicine, Mansoura University, Dakahleya, Egypt; ^10^Department of Internal Medicine, Rheumatology and Clinical Immunology Unit, Faculty of Medicine, Cairo University, Cairo, Egypt; ^11^Faculty of Medicine, Newgiza University, Giza, Egypt; ^12^Department of Neurology, Faculty of Medicine, Mansoura University, Dakahleya, Egypt; ^13^Department of Neurology, Faculty of Medicine, Alexandria University, Alexandria, Egypt; ^14^Department of Neurology, Mansoura International Hospital, Dakahleya, Egypt; ^15^Forensic Medicine and Clinical Toxicology Department, Faculty of Medicine, Mansoura University, Dakahleya, Egypt; ^16^Department of Neuropsychiatry, Faculty of Medicine, Kafr El Sheikh University, Egypt

**Keywords:** Parkinson’s disease, genetics, APOE, KASP, Egyptian

## Abstract

**Background:**

The apolipoprotein E (APOE) gene, encompassing three alleles (ε2, ε3, ε4), is a critical player in lipid metabolism and has been extensively studied for its role in neurodegenerative diseases. This study examines APOE genetic variability and its association with PD in an Egyptian cohort.

**Methods:**

A total of 891 participants, including 422 PD patients and 469 healthy controls, were included in this study. APOE genotyping was performed using Kompetitive Allele Specific PCR (KASP) to detect the rs429358 and rs7412 SNPs, which define the APOE alleles. APOE alleles were categorized based on the genotypes into ε2, ε3, and ε4 groups. Clinical assessments of PD patients included age at onset, disease severity (MDS-UPDRS), and demographic factors. Statistical analyses compared APOE distributions between PD and control groups and examined associations with clinical variables.

**Results:**

The ε3 allele was the most prevalent in the cohort (77.3%), aligning with global and African trends. The ε2 allele was observed in 11.4%, and the ε4 allele in 11.3%, with both frequencies being lower than reported African estimates. The ε3/ε3 genotype was predominant in both PD patients (72.51%) and controls (72.07%). The ε4/ε4 genotype was absent in PD cases and rare among controls (0.64%). No significant association was found between APOE genotypes and PD risk, age at onset, or disease severity.

**Conclusion:**

Our findings do not support a significant role for APOE in PD susceptibility or severity in Egyptians.

## Introduction

The apolipoprotein E (APOE) gene, located on chromosome 19q13.2, comprises three common polymorphic alleles (ε2, ε3, ε4), resulting in six possible genotypes (ε2/ε2, ε2/ε3, ε2/ε4, ε3/ε3, ε3/ε4, ε4/ε4). APOE is crucial for lipid metabolism and is implicated in various neurodegenerative disorders, most notably Alzheimer’s disease (AD). The ε4 allele is recognized as a significant genetic risk factor for late-onset AD, associated with increased disease susceptibility and earlier onset, while the ε2 allele may provide some protective effects (Panza et al., 2012; Chartier-Hariln et al., 1994; Lambert et al., 2013). Such modulatory effects of APOE are strongly linked to its alleles differential modulation of amyloid-*β* metabolism, aggregation, and clearance, in addition to influencing tau pathology, neuroinflammation, and neuronal signaling (Kanekiyo et al., 2014; Baek et al., 2020).

Beyond AD, it has been suggested that intracellular *α*-synuclein contributes to Parkinson’s Disease (PD) pathology by partially activating extracellular signaling pathways involving APOE (Gallardo et al., 2008). The mechanisms underlying APOE’s potential involvement in PD, however, are less understood and likely distinct from those in AD, reflecting the different core pathologies. Several hypotheses attempt to bridge APOE function with PD pathophysiology. These include suggestions that APOE isoforms may differentially affect *α*-synuclein aggregation (Emamzadeh et al., 2016), propagation, or clearance (Kang et al., 2022; Liampas et al., 2024; Zhao et al., 2020), modulate microglial activation and neuroinflammatory responses in the context of synucleinopathy (Zhang et al., 2023), or influence lipid transport and cholesterol metabolism critical for dopaminergic neuron integrity and function (Fernández-Calle et al., 2022). Despite these plausible biological connections, establishing a definitive link between APOE variants and PD risk or progression has proven challenging.

Unlike in AD, the role of APOE in PD, especially the ε4 allele, remains less clearly defined and studies are yielding contradicting findings (Federoff et al., 2012; Li et al., 2018). Recent large genome-wide association studies (GWAS) on individuals of Northern European descent revealed no significant link between APOE genotype and PD status or age at onset. However, meta-analyses that included data from diverse ethnic groups—such as Europeans, Asians, and Latin Americans—demonstrate that the link between APOE genotype and PD risk may be ancestry dependent (Li et al., 2018; Sun et al., 2019).

Furthermore, the association between APOE and disease severity in PD is less straightforward. Although animal research suggests that the APOE genotype influences motor function in PD, with ε4 carriers showing worse motor performance and ε2 carriers experiencing less motor dysfunction (Davis et al., 2020), this association has not been established in clinical research (Kapan et al., 2023; Jo et al., 2021). This discrepancy highlights the need for further investigation into how APOE may differentially affect motor outcomes.

Despite considerable research on the APOE gene and its role in neurodegenerative diseases, studies focusing on PD in populations from the Middle East and Africa, including Egyptians, remain limited. This study aims to characterize the genetic variability of the APOE gene in Egyptians with and without PD. Additionally, it investigates APOE’s influence on genetic susceptibility to PD and explore how APOE variants may affect the age at onset and disease severity of PD within the study population.

## Methods

The present study received ethical approval from the American University in Cairo review board (AUC-IRB) in Egypt, with the following reference numbers: Ethics Approval #2021–2022-058 and #2021–2022-203. Additionally, the ethical committee of University College London granted approval under REC #22/NE/0080. Written informed consent was obtained from all participants, in compliance with the Declaration of Helsinki and the Common Rule.

### Participant recruitment and clinical assessments

A total of 891 Egyptians, comprising 422 PD patients and 469 unrelated healthy controls, were recruited through the Egyptian Network of Neurodegenerative Diseases (ENND) as a collaboration with International Parkinson’s Disease Genome Consortium- Africa (IPDGC-Africa). PD was diagnosed by neurologists using the UK Brain Bank Criteria (Hughes et al., 1992) and/or the criteria established by the Movement Disorder Society Task Force (Postuma et al., 2015). Basic demographic information, including age and gender, was collected from all subjects. Clinical information for PD cases included age at disease onset, age at diagnosis, family history, and history of consanguinity. Additionally, the Movement Disorder Society Unified Parkinson’s Disease Rating Scale (MDS-UPDRS) were assessed in all PD cases.

### APOE genotyping

DNA was extracted from 10 mL samples of either venous whole blood or saliva using established protocols. Genotyping was performed using the Kompetitive Allele Specific Polymerase Chain Reaction (KASP) assay (LGC Genomics, Herts, United Kingdom), following the method outlined in Rizig et al. (2021). Two single-nucleotide polymorphisms (SNPs) from the APOE gene, specifically rs429358 and rs7412, were selected for genotyping. These SNPs are the primary genetic markers used to determine the APOE isoforms (ε2, ε3, ε4). Functionally, these SNPs influence the structure and function of the APOE protein, which plays a crucial role in neurodegeneration (Corbo and Scacchi, 1999). Both SNPs have validated KASP assays available, offering an efficient, cost-effective, and reliable method for genotyping our cohort.

### Data analysis

APOE alleles were categorized based on the genotypes as follows: individuals with the ε2/ε2 genotype were assigned to the ε2 group; those with either ε2/ε3 or ε3/ε3 genotypes were classified into the ε3 group; and individuals with the ε2/ε4, ε3/ε4, or ε4/ε4 genotypes were grouped under the ε4 category.

## Results

Cohort characteristics is summarized in Table 1.

**Table 1:** Demographic and clinical characteristics of PD and controls.

Characteristics	PD			Controls		
	Total	Male	Female	Total	Male	Female
Study participants n, (%)	422 (100)	274 (64.93)	148 (35.07)	469	173 (36.89)	296 (63.11)
Age at Study (y)	61.35 ± 10.32	61.38 ± 9.97	61.28 ± 10.96	44.53 ± 13.86	49.58 ± 13.64	41.57 ± 13.14
Age at onset (y)	54.08 ± 9.23	54.4 ± 9.25	53.48 ± 9.19	**–**	–	–
Age at Diagnosis (y)	54.65 ± 9.33	54.91 ± 9.35	54.17 ± 9.32	–	–	–
Duration of PD (y)	7.27 ± 6.06	6.98 ± 5.99	7.8 ± 6.17	–	–	–
MDS-UDPRS
	96.26 ± 54.42	98.41 ± 53.36	92.28 ± 56.28	–	–	–
History of consanguinity, n (%)	17 (4)	8 (2.9)	9 (6.1)	–	–	–
Family History, n (%)	57 (13.5)	32 (11.7)	25 (16.9)	–	–	–

Data presented as mean ± standard deviation, except where indicated. A significantly higher proportion of controls were females (*p* < 0.001), while a significantly higher proportion of PD cases were males (*p* < 0.001). Mean age at the time of the study differed significantly between PD cases and controls, both overall and by gender (*p* < 0.001). Among PD cases, mean age did not differ significantly by gender (*p* = 0.93), age of onset (*p* = 0.33), age at diagnosis (*p* = 0.44), PD duration (*p* = 0.18), or UPDRS score (*p* = 0.27). There was no significant difference in the proportion of males and females with family history of PD (*p* = 0.14) or history of consanguinity (*p* = 0.13) among PD cases.

### APOE allelic and genotypic frequency proportions in Egyptians with PD and controls

**Figure 1:**
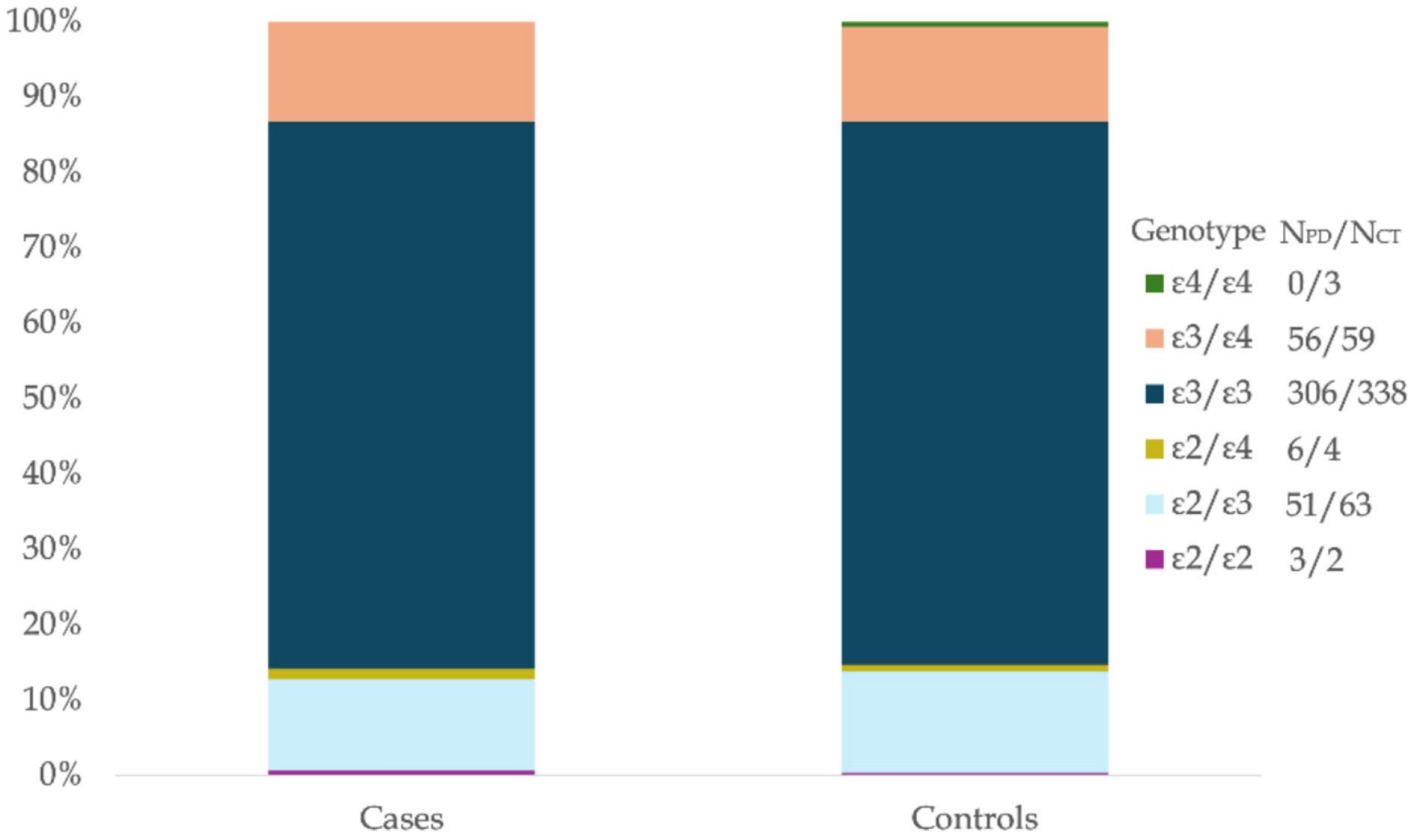
APOE genotype distribution in Egyptians with PD and controls. Percentage distribution of APOE genotypes in Egyptian Parkinson’s Disease (PD) patients (Cases, *N* = 422) and ethnically matched healthy controls (Controls, *N* = 469). The stacked bars illustrate the relative frequencies of the ε2/ε2, ε2/ε3, ε2/ε4, ε3/ε3, ε3/ε4, and ε4/ε4 genotypes within each group. Absolute counts for each genotype are indicated in the legend for the cases and control groups.

In this study, the allele frequencies of APOE among all participants (n = 891) were as follows: ε3 (77.257%), ε4 (11.327%), and ε2 (11.416%). The genotypic frequencies were 72.28% for ε3/ε3, 0.34% for ε4/ε4, and (0.56%) for ε2/ε2 among the homozygotes, while the heterozygous frequencies were ε3/ε4 (12.91%), ε2/ε3 (12.79%) and ε2/ε4 (1.12%). The APOE allelic and genotypic distributions observed in this Egyptian cohort were generally consistent with reports from similar populations (Figure 1; Tables 2, 3).

**Table 2:** APOE allele distribution in Egyptians with PD and controls in comparison to other normal global and ethnic populations.

Allele n (%)	All, *N* = 891	PD, *N* = 422	Controls *N* = 469	Global (Abondio et al., 2019)	Africans (Abondio et al., 2019)	Europeans (Corbo and Scacchi, 1999)	Asians (Corbo and Scacchi, 1999)	Native Americans (Corbo and Scacchi, 1999)	Oceanians (Corbo and Scacchi, 1999)
ε2	129 (11.416)	60 (11.215)	69 (11.597)	0–38	2.7–11.6	4.4–11.9	0.4–14.0	0.0–1.4	0.0–14.5
ε3	873 (77.257)	413 (77.197)	460 (77.311)	48–94	53.6–85	64–89.8	62–87.0	72–91.1	48.6–74
ε4	128 (11.327)	62 (11.589)	66 (11.092)	3–41	14.3–40.7	6.8–31	7.1–24	8.9–28	26–68

Denominator for allele frequencies is total allele count (Egyptian cohort present study: all = 1,130, PD = 535, controls = 595). No significant difference in allele frequencies in PD versus controls in this study. Odds ratios (95% CI) PD versus controls [ε2: 0.96 (0.67–1.39), *p* = 0.84; ε3: 0.99 (0.75–1.31), *p* = 0.96; ε4: 1.05 (0.73–1.52), *p* = 0.79].

**Table 3:** APOE genotype distribution in Egyptians with PD and controls in comparison to other normal global and ethnic populations.

Genotype	All participants *N* = 891	PD *N* = 422	Controls *N* = 469	Global (Qin et al., 2021)	Black (Qin et al., 2021)	Whites (Qin et al., 2021)
ε2/ε2	5 (0.56)	3 (0.71)	2 (0.43)	0.53	1.23	0.5
ε2/ε3	114 (12.79)	51 (12.09)	63 (13.43)	11.99	13.38	12.71
ε2/ε4	10 (1.12)	6 (1.42)	4 (0.85)	1.78	3.44	2.21
ε3/ε3	644 (72.28)	306 (72.51)	338 (72.07)	65.68	47.86	60.16
ε3/ε4	115 (12.91)	56 (13.27)	59 (12.58)	18.6	30.65	22.43
ε4/ε4	3 (0.34)	0 (0)	3 (0.64)	1.41	3.44	1.99

No significant difference in genotype frequencies in PD versus controls in this study. *p*-value for comparison of genotype frequencies in present study Fischer (*p* > 0.05). Genotype frequencies for controls, as reported by Qin et al (Qin et al., 2021),. were categorized globally and by race (‘Black’ and ‘White’) according to the classifications outlined in the publication.

### Association between APOE genotypes and PD age of onset, and disease severity

The link between APOE genotypes and Parkinson’s disease (PD) age of onset was assessed using linear regression. The analysis revealed that APOE genotypes explained only a small proportion of the variance in age of onset (R^2^ = 0.021), and this association was not statistically significant (F-statistic = 1.787, *p* = 0.114). Similarly, APOE genotypes were not significant predictors of disease severity, as measured by the MDS-UPDRS scores. In contrast, age, age of onset, and disease duration emerged as significant predictors of disease severity, underscoring their importance in the progression of PD.

## Discussion

A detailed comparison of the herein communicated APOE genotype distribution findings with those from previous studies conducted in Egypt underscores the contributions of the present study (Table 4). Notably, our study represents the largest cohort to date examining APOE variants in Egyptian PD patients (*N* = 422) and controls (*N* = 469), significantly exceeding the sample size of the only other published study specifically focusing on PD in Egyptians (*N* = 55 patients, *N* = 30 controls) by Fahmy et al. Furthermore, our cohort encompasses a broader geographical representation, including participants from 16 different governorates, in contrast to previous studies which were often restricted to single locales such as Cairo, Mansoura, or Ismailia. This enhanced sample size and diversity strengthen the reliability and generalizability of our findings for the Egyptian population.

**Table 4:** Comparison of the *APOE* genotypes distribution with different diseases among Egyptians.

(Reference, year); location	Disease	Patients, n (%)							Healthy controls, n (%)						
		N	Genotypes, n (%)						N	Genotypes, n (%)					
			ɛ3/ɛ3	ɛ2/ ɛ2	ɛ2/ ɛ3	ɛ2/ ɛ4	ɛ3/ ɛ4	ɛ4/ ɛ4		ɛ3/ ɛ3	ɛ2/ ɛ2	ɛ2/ ɛ3	ɛ2/ ɛ4	ɛ3/ ɛ4	ɛ4/ ɛ4
Our Study (Current Study); 16 governates	PD	422	306 (72.51)	3 (0.71)	51 (12.09)	6 (1.42)	56 (13.27)	0 (0)	469	338 (72.07)	2 (0.43)	63 (13.43)	4 (0.85)	59 (12.58)	3 (0.64)
Fahmy et al (Fahmy et al., 2024).; Cairo	PD	55	8 (14.5)	1 (1.8)	14 (25.5)	4 (7.3)	24 (43.6)	4 (7.3)	30	3 (10)	1 (3.3)	6 (20)	9 (30)	11 (36.7)	0 (0)
Taha et al (Taha et al., 2024).; Cairo	MI	100	36 (36)	0 (0)	0 (0)	0 (0)	64 (64)		100	60 (60)	0 (0)	0 (0)	0 (0)	40 (40)	
Amer et al (Amer et al., 2021).; Cairo	AD	32	19 (60)	0 (0)	0 (0)	0 (0)	10 (31.4)	3 (8.6)	24	20 (82.9)	0 (0)	4 (17.1)	0 (0)	0 (0)	0 (0)
	MCI	32	26 (80)	0 (0)	1 (2.9)	0 (0)	5 (17.1)	0 (0)							
Galal et al (Galal et al., 2021).; Mansoura	T2DM + obesity	100	37 (37)	32 (32)	5 (5)	4 (4)	6 (6)	16 (16)	100	58 (58)	12 (12)	9 (9)	7 (7)	10 (10)	4 (4)
	T2DM	100	46 (46)	18 (18)	6 (6)	5 (5)	12 (12)	13 (13)							
	Obesity	100	52 (52)	24 (24)	14 (14)	2 (2)	5 (5)	3 (3)							
Hemeda et al (Hemeda et al., 2021).; Cairo	HCV	100	6 (6)	15 (15)	52 (52)	21 (21)	0 (0)	6 (6)	__	__	__	__	__	__	__
Ramadan et al (Ramadan et al., 2019).; Cairo	AD	53	30 (56.6)	0 (0)	2 (3.8)	0 (0)	12 (22.6)	9 (17)	100	87 (87)	0 (0)	5 (5)	0 (0)	8 (8)	0 (0)
Hassan et al (Hassan et al., 2019).	Metabolic syndrome	44	16 (36.4)	0 (0)	0 (0)	4 (9.1)	20 (45.5)	4 (9.1)	156	68 (43.6)	0 (0)	0 (0)	12 (7.7)	36 (23.1)	40 (25.6)
Arafa et al (Arafa et al., 2018).; Mansoura	CHD	100	59 (59)	0 (0)	9 (9)	0 (0)	32 (32)	0 (0)	100	73 (73)	0 (0)	13 (13)	0 (0)	14 (14)	0 (0)
Gomaa et al (Gomaa et al., 2018).; Giza	HCV	125	25 (20)	0 (0)	0 (0)	0 (0)	99 (79)	1 (0.8)	120	6 (5)	0 (0)	0 (0)	0 (0)	111 (92.5)	3 (2.5)
Fawzy et al (Fawzy et al., 2017).; Ismailia	CAD	23	15 (65.2)	1 (4.3)	3 (13.0)	1 (4.3)	3 (13.0)	0 (0)	34	10 (29.4)	0 (0.0)	20 (58.8)	2 (5.9)	2 (5.9)	0 (0.0)
Hassan NE (Hassan, 2016)	Obesity	66	22 (33.3)	0 (0)	0 (0)	8 (12.1)	22 (33.3)	14 (21.2)	36	18 (50.0)	0 (0)	0 (0)	8 (22.2)	2 (5.6)	8 (22.2)
	Obesity + Visceral obes.	56	22 (39.3)	0 (0)	0 (0)	12 (21.4)	8 (14.3)	14 (25.0)							
El-Lebedy et al (El-Lebedy et al., 2016).; Cairo	T2DM + CVD	100	65 (65)	0 (0)	10 (10)	0 (0)	25 (25)	0 (0)	84	66 (78.6)	0 (0)	11 (13.1)	0 (0)	7 (8.3)	0 (0)
	T2DM	100	80 (80)	0 (0)	8 (8)	0 (0)	12 (12)	0 (0)							
Atta et al (Atta et al., 2016).; Beni Suef	T2DM	45	12 (26.7)	0 (0)	12 (26.7)	12 (26.7)	9 (20)	0 (0)	45	30 (66.7)	0 (0)	3 (6.7)	3 (6.7)	9 (20)	0 (0)
	T2DMN	45	0 (0)	0 (0)	27 (60)	18 (40)	0 (0)	0 (0)							
Mahmoud et al (Mahmoud et al., 2016).; Sohag	ACS	200	124 (62)	2 (1)	21 (10.5)	7 (3.5)	42 (21)	4 (2)	100	70 (70)	2 (2)	20 (20)	1 (1)	6 (6)	1 (1)
El-Tagui et al (El-Tagui et al., 2013).; Cairo	β-thalassemia	50	28 (56)	0 (0)	11 (22)	0 (0)	6 (12)	5 (10)	50	39 (78)	1 (2)	4 (8)	0 (0)	6 (12)	0 (0)
Halim et al (Halim et al., 2012).; Menofiya	CAD + T2DM	35	18 (51.4)	6 (17.1)	5 (14.3)	0 (0)	6 (17.1)	0 (0)	35	31 (88.6)	0 (0)	2 (5.7)	0 (0)	2 (5.7)	0 (0)

Data is presented as numbers (percentage). PD, Parkinson’s Disease; MI, Myocardial Infarction; AD, Alzheimer’s disease; MCI, Mild cognitive impairment; T2DM, type 2 diabetes mellitus; CHD, coronary heart disease; HCV, hepatitis C virus; CVD, cardiovascular disease; T2DMN, type 2 diabetes mellitus with nephropathy; ACS, acute coronary syndrome; CAD, coronary artery disease.

In terms of genotype distribution among PD patients, our results show a higher prevalence of the ε3/ε3 genotype (72.51%) compared to the Fahmy et al. study (14.5%). Conversely, the frequencies of the ε3/ε4 (13.27%) and ε4/ε4 (0%) genotypes in our PD cohort were lower than those previously reported (43.6 and 7.3%, respectively). Differences are also observed when comparing our control group’s genotype frequencies (e.g., ε3/ε3: 72.07%, ε3/ε4: 12.58%, ε4/ε4: 0.64%) to those in the aforementioned study’s controls (ε3/ε3: 10%, ε3/ε4: 36.7%, ε4/ε4: 0%) and other Egyptian studies focusing on different diseases such as AD, Myocardial Infarction (MI), or Type 2 Diabetes Mellitus (T2DM). These variations underscore the potential influence of cohort size, geographical location, and the specific disease context on APOE genotype distributions within the Egyptian population.

On the other hand, the APOE allelic and genotypic frequencies in our cohort align broadly with the distributions reported in populations of African ancestry, with some notable distinctions. Corroborating other studies, the ε3 allele was the most frequent, detected in ~ 77.3% of all participants, placing it within the mid-range of reported rates in African populations (53.6–85%) and consistent with other ethnic groups worldwide (48–94%; Abondio et al., 2019). The ε2 allele was present in 11.4% of the entire cohort, a prevalence that is consistent with previous reports from Africa, albeit at the higher end of the reported range (2.7–11.6%), and comparable to global frequencies (Abondio et al., 2019). In contrast, the frequency of the ε4 allele was lower than the African estimates (14.3–40.7; Abondio et al., 2019), with only 11.3% of our cohort carrying this allele. Within genotypic distributions, ε3/ε3 was the most prevalent in both the PD group (72.51%) and controls (72.07%), aligning with earlier studies (Galal et al., 2021; Ramadan et al., 2019) and mirroring global trends where the ε3 allele predominates (Qin et al., 2021). The ε2/ε2 genotype, observed in 0.71% of PD patients and 0.43% of controls, falls within global estimates, but lower than the frequencies typically observed in individuals of black ethnicity (Qin et al., 2021). The ε2/ε4 and ε3/ε4 combinations were relatively uncommon, with the ε3/ε4 genotype showing comparable frequencies between PD patients (13.27%) and controls (12.58%), which are lower than both global and regional estimates. Further, the ε4/ε4 genotype was not observed in PD cases and was found in only 0.64% of controls, a frequency much lower than the global estimates regardless of the ethnicity (Qin et al., 2021).

Significant gene flow from the Near East and Mediterranean/European populations, which generally have lower ε4 frequencies compared to ancestral Sub-Saharan African populations (Corbo and Scacchi, 1999), could have substantially shaped the Egyptian gene pool over millennia (Bassyouni et al., 2023). Additionally, while ε4 is the ancestral human allele and may have offered advantages in past environments (e.g., related to diet or pathogen resistance; Fujioka et al., 2013), the long history of agriculture in the Nile Valley might have favored the ε3 allele. Furthermore, the shift toward modern diets could potentially exert negative selective pressure on ε4 due to its association with increased risk for cardiovascular and metabolic diseases in contemporary settings (Huebbe and Rimbach, 2017). Therefore, the current ε4 frequency likely reflects a blend of ancient African history diluted by substantial admixture from regions with lower ε4 prevalence, and possibly finetuned by selective pressures related to historical changes in diet and lifestyle.

Regarding the secondary objectives of our study, we found no significant association between APOE genotypes and the risk of PD. This is consistent with prior research, which similarly reported no notable differences in APOE distribution, including ε4 carrier rates, between PD patients and healthy controls (Federoff et al., 2012; Okubadejo et al., 2022; Williams-Gray et al., 2009). Furthermore, we observed no significant relationship between APOE alleles or genotypes and the age of onset or disease severity of PD. The lack of association with disease severity, despite suggestive findings in some animal models (Davis et al., 2020; Fyfe, 2020), might stem from several factors in human studies: the complex interplay between genetic predisposition and diverse environmental influences throughout life, potential modification of APOE effects by other genetic loci not assessed here, or inherent differences between preclinical models and the multifaceted pathophysiology of human PD. Biologically, while APOE is crucial for lipid transport and neuronal maintenance (Yang et al., 2023; Husain et al., 2021), its specific role in the dopaminergic pathways primarily affected in PD may be less pronounced compared to its established impact in Alzheimer’s pathology, potentially explaining the divergence from findings in AD and the lack of strong association signals in many PD cohorts globally (Federoff et al., 2012; Okubadejo et al., 2022; Kurz et al., 2009; Wenjuan et al., 2016), including ours.

Notwithstanding the above, we acknowledge several limitations in our study. While 891 participants represent a relatively large dataset for a country-specific genetic study, this size may be insufficient to detect small effect sizes, or gene–gene/gene–environment interactions, particularly for rare alleles such as ε2 or ε4. This may partly explain the absence of significant associations between APOE and PD risk or severity in our study. Furthermore, our sample did not include any PD cases homozygous for the ε4 allele, which may have further limited our ability to assess its potential impact on disease risk or severity. For assessing PD severity, we relied on the total MDS-UPDRS score, as detailed sub-scores were unavailable. This limitation restricted our ability to explore more nuanced associations between APOE genotypes and specific symptom domains. Additionally, we were unable to examine the potential link between APOE and specific non-motor features like cognitive impairment, where APOE effects might be more relevant. Therefore, future studies with larger sample sizes and more detailed phenotypic data are needed to clarify APOE’s role in PD.

In addition, APOE is likely only one piece of a complex genetic landscape. Exploring alternative or complementary genetic pathways is crucial. Other PD-associated genes such as LRRK2, GBA, SNCA, and MAPT should be investigated in the Egyptian population to identify more relevant genetic risk factors. Furthermore, environmental contributors—including pesticide exposure, rural residency, dietary habits, and access to healthcare—may interact with genetic predispositions in shaping PD risk. Incorporating these variables into future studies may yield a more comprehensive understanding of PD etiology in this population.

Despite these limitations, our study provides valuable insights into the distribution of APOE genotypes in Egyptians with PD and contributes to the broader body of knowledge on genetic risk factors for PD in North African populations. Further research is needed to explore the role of APOE and other genetic and environmental factors in PD, with larger sample sizes and more comprehensive data collection.

## Conclusion

This study provides the largest exploration to date of APOE genetic variability in an Egyptian cohort with PD, offering new insights into the distribution of APOE alleles and genotypes in populations of Middle Eastern and African descent, which are traditionally underrepresented in genetic studies. The findings suggest that APOE allelic and genotypic distributions in Egyptians broadly align with African and global trends, with some distinctions, particularly in the lower frequency of the ε4 allele and the strikingly low prevalence of the ε4/ε4 genotype. Importantly, our analysis found no significant association between APOE genotypes and the overall risk of PD, nor with the age of onset or severity of the disease within our cohort’s statistical power. While these results align with some large-scale studies in other populations suggesting APOE may not be a major driver of PD risk or progression, it is important to acknowledge that this does not preclude potential influences on other specific aspects of the disease not fully captured here, such as cognitive decline, or potential interactions with other genetic or environmental factors unique to this population. Therefore, future research should focus not only on larger sample sizes and diverse cohorts from the Middle East and Africa but also incorporate longitudinal study designs to track progression, detailed cognitive assessments stratified by genotype, and investigations into potential gene–environment interactions. Such focused approaches are warranted to fully elucidate the potentially subtle or context-dependent roles of APOE and to enhance our understanding of the complete genetic architecture contributing to PD in these populations.

## Data Availability

Anonymized genotyping and clinical data can be provided to bone fide researchers upon request from the corresponding author(s).
